# Immune Cytolytic Activity for Comprehensive Understanding of Immune Landscape in Hepatocellular Carcinoma

**DOI:** 10.3390/cancers12051221

**Published:** 2020-05-13

**Authors:** Hideo Takahashi, Tsutomu Kawaguchi, Li Yan, Xuan Peng, Qianya Qi, Luc G.T. Morris, Timothy A. Chan, Allan Tsung, Eigo Otsuji, Kazuaki Takabe

**Affiliations:** 1 Department of Surgical Oncology, Roswell Park Comprehensive Cancer Center, Buffalo, NY 14263, USA; hideo.takahashi@roswellpark.org (H.T.); t-kwgch@koto.kpu-m.ac.jp (T.K.); 2 Department of Surgery, Kyoto Prefectural University of Medicine, Kyoto 602-8566, Japan; otsuji@koto.kpu-m.ac.jp; 3 Department of Biostatistics and Bioinformatics, Roswell Park Comprehensive Cancer Center, Buffalo, NY 14263, USA; li.yan@roswellpark.org (L.Y.); xuanpeng@buffalo.edu (X.P.); qianya.qi@buffalo.edu (Q.Q.); 4 Immunogenomics and Precision Oncology Platform, Memorial Sloan Kettering Cancer Center, New York, NY 14263, USA; morrisl@mskcc.org (L.G.T.M.); chant@mskcc.org (T.A.C.); 5 Department of Surgery, Memorial Sloan Kettering Cancer Center, New York, NY 10065, USA; 6 Human Oncology and Pathogenesis Program, Memorial Sloan Kettering Cancer Center, New York, NY 10065, USA; 7 Department of Radiation Oncology, Memorial Sloan Kettering Cancer Center, New York, NY 10065, USA; 8 Center for Immunotherapy and Precision Immuno-Oncology, Cleveland Clinic, Cleveland, OH 44195, USA; 9 Lerner Research Institute and Taussig Cancer Center, Cleveland Clinic, Cleveland, OH 44195, USA; 10 Division of Surgical Oncology, Department of Surgery, The Ohio State University Wexner Medical Center, Columbus, OH 43210, USA; Allan.Tsung@osumc.edu; 11 Department of Surgery, University at Buffalo Jacobs School of Medicine and Biomedical Sciences, the State University of New York, Buffalo, NY 14260, USA; 12 Department of Gastroenterological Surgery, Yokohama City University Graduate School of Medicine, Yokohama 236-0004, Japan; 13 Department of Surgery, Niigata University Graduate School of Medical and Dental Sciences, Niigata 951-8510, Japan; 14 Department of Breast Surgery and Oncology, Tokyo Medical University, Tokyo 160-8402, Japan

**Keywords:** hepatocellular carcinoma, cytolytic activity, tumor infiltrating lymphocyte, TCGA

## Abstract

Cytolytic activity score (CYT), defined by granzyme A and perforin expression, is a useful marker for underlying immunity. We hypothesized that CYT-high hepatocellular carcinomas (HCCs) have stronger immunogenicity and favorable tumor microenvironments, which would result in better clinical outcomes, using the cancer genome atlas (TCGA) cohort with 371 patients with HCC. We found CYT-high HCCs were associated with higher expressions of the apolipoprotein B mRNA editing enzyme catalytic polypeptide-like 3 (APOBEC3), well-known mutagenic enzymes. Further, higher numbers of anti-cancer immune cells, such as CD8+ T cells and M1 macrophages, were infiltrated in CYT-high HCCs. Major T cell exhaustion markers were expressed significantly higher in CYT-high HCCs, likely as a negative feedback loop. Additionally, CYT-high HCCs strongly enriched gene sets related with enhanced immune activity. With strong immunity, patients with CYT-high HCCs had significantly longer disease-specific survival (DSS) and overall survival (OS) (*p* = 0.03 and <0.01). Furthermore, when the OS is stratified by exhaustion marker expressions, the CYT-high/exhaustion-low group had the best and CYT-low/exhaustion-high groups had the worst OS. Lastly, high CYT was an independent protective factor for prognosis. In conclusion, CYT-high HCCs were associated with enhanced immunity and better survival. Our findings suggest that proper identification of tumor-immune microenvironments could stratify the patients for appropriate treatments.

## 1. Introduction

T cells develop stepwise and progressive loss-of-effector functions by expressing T cell exhaustion markers or “brakes to the T cells”. The identification of T cell exhaustion markers and subsequent intense investigation in cancer immunity lead to the development of immune checkpoint inhibitors (ICIs), which have revolutionized cancer treatment. ICIs, such as anti-cytotoxic T-lymphocyte-associated protein 4 (CTLA4), anti-programmed death protein-1 (PD-1), or anti-programmed death-ligand 1 (PD-L1) antibodies, target T cell exhaustion markers and enhance antitumor immunity by reversing the exhaustion of cytotoxic T cells, also known as “taking off the brake of the T cells” [1,2,3]. Emerging evidence has revealed that anticancer efficacy of ICIs is dependent on the characteristics of the tumor microenvironment (TME), as they require functional cytotoxic lymphocytes. The TME with abundant tumor infiltrating lymphocytes (TILs), such as cytotoxic T cells, natural killer (NK) cells, and macrophage M1, is referred as “hot” or “immune-inflamed” in contrast to the “cold” TME with immunosuppressive lymphocytes, such as regulatory T cells [4]. It is conceivable that ICIs are highly effective in the “hot” TME, since the high density of TILs recognize ample tumor specific antigens with their diverse receptors. Similarly, a few predictive biomarkers for ICIs have been identified, such as a high expression of PD-L1, mismatch repair deficiency, and increased tumor mutation load [5,6,7]. Increased mutation in cancer is caused by several mechanisms, including exogenous mutagens and endogenous processes. While exogenous mutagens are consisted of exposure to UVA, UVB, or toxic substances in smoking, endogenous processes include mismatch repair deficiency and excessive Apolipoprotein B mRNA editing enzyme catalytic polypeptide-like 3 (APOBEC3) activities [8,9,10]. On the other hand, negative predictors for ICIs, so-called “immune exclusion”, have also been investigated in various cancers. Beta-catenin pathway activation or an increased *CTNNB1* score resulted in T cell exclusion and demonstrated resistance to ICIs in melanomas and hepatocellular carcinomas (HCCs) [7,11]. Despite these emerging evidences, however, we continue to lack reliable predictors for ICIs in order to stratify the patients for appropriate immunotherapy based on the immune landscape.

Hepatocellular carcinoma (HCC) is the fourth-most common cause of cancer-related deaths in the world and is the most common type of primary liver tumor [12,13,14]. The prognoses of patients with advanced HCC remain dismal, despite multiple treatment options, including liver resection, thermal ablation, trans-arterial chemo or radio-embolization, liver transplant, and systemic chemotherapy with tyrosine kinase inhibitors [15,16,17]. Hence, ICIs have been recently investigated in HCCs by several clinical trials exploiting from the excellent results in other cancers [1,2,3,17,18,19]. These trials revealed that, although ICIs did achieve durable responses in a small subset of patients, the overall response rate was disappointingly low with 15–17% [3,18,19]. Additionally, although PD-L1 expression was suggested as a potential predictor of a response to ICIs in HCC, it remains imperative and in urgent need to define better biomarkers in order to identify the respondents, enabling adequate patient selection for ICIs [5,6,7,20,21,22].

Rooney et al. reported a quantitative measure of immune cytolytic activity (CYT) based on the expression levels of granzyme A (GZMA) and perforin 1 (PRF1), which were significantly upregulated with cytotoxic T cell activation [23]. Cytotoxic T cells primarily kill cancer cells using these two enzymes; perforin is an enzyme forming pores on target cell membranes, through which granzymes enter and activate caspase-independent apoptosis [23]. The density of TILs by immunohistochemistry (IHC) has been known as a strong predictor of favorable outcomes in various cancers, even before the era of ICIs, independent from tumor histology, metastatic status, or tumor stage [24,25]. However, as GZMA and PRF1 are very specific to cytotoxic T cells in heterogeneous tumor samples, CYT measures anticancer immunity through gene expression in lieu of predicting T cell function simply from the density of TILs by IHC [20,26,27]. The greatest strength of CYT is its wide availability and reproducibility without significant costs, as it requires only the RNA sequences of GZMA and PRF1 from the tumors.

Here, we hypothesized that HCCs with high CYT have stronger immunogenicity and the favorable immune TME that would result in better tumor biology and clinical outcomes.

## 2. Results

### 2.1. Patient Demographics

Among the cancer genome atlas (TCGA) liver hepatocellular carcinoma (LIHC) cohort, 185 patients (49.9%) were CYT-high, and 186 patients (50.1%) were CYT-low when the cut-off was determined by the median of CYT. Gene expressions of granzyme A and perforin were distributed normally in this cohort (Figure S1a). CYT was found to be lower in HCC compared to the normal liver tissue (*p* = 0.019, Figure S1b). This is likely due to less stromal cells, including immune cells in the TME. In general, immune systems in cancer tissues are thought to be less active compared to the normal liver tissue. There were no significant differences in clinical demographics between CYT-high and CYT-low groups, except the presence of TILs by immunohistochemistry (IHC) (*p* < 0.0001, Table 1). Although the degree of cirrhosis was reported to affect immune function significantly [28], the extent of cirrhotic change of the background liver was not significantly different between the groups (Table 1). Additionally, CYT was not significantly different based on the underlying inflammation extent as well (Figure S1c). Therefore, the TCGA LIHC cohort did not contain any treatment information. 

### 2.2. CYT-High Hccs were Associated with Significantly Higher Gene Expressions of Apolipoprotein B mrna Editing Enzyme Catalytic Polypeptide-Like 3 (APOBEC3) Family Members

**Figure 1:**
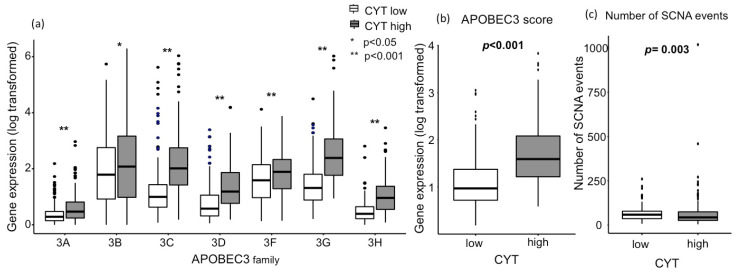
Association between the CYT and APOBEC3 family gene expression, as well as a number of related SCNA events. (**a**) Individual APOBEC3 family gene expression differences between CYT groups (*p* < 0.05 on APOBEC3B and *p* < 0.001 on the rest of APOBEC3 family). (**b**) Combined APOBEC3 score (*p* < 0.001). (**c**) The number of APOBEC3 family gene-related SCNA events (*p* = 0.003). There are 185 and 186 patients in CYT-high and low groups, respectively. All comparisons were performed using the Mann-Whitney test. CYT, cytolytic activity score; APOBEC3, apolipoprotein B mRNA editing enzyme catalytic polypeptide-like 3; and SCNA, somatic copy number alterations.

Association between the high mutation load and the highest response rate with ICIs has been observed in tumors with a high mutation burden, such as melanoma and lung cancer [1,5,6]. Cytolytic activity was reported to positively correlate with neoantigen loads and the mutation load in multiple tumor types [23]. APOBEC3 family members are mutagenic enzymes that cause DNA mutations and RNA editing in various cancers and fuel cancer heterogeneity [8,9]. Hence, we hypothesized that gene expressions of APOBEC3 members are elevated in CYT-high HCCs, leading to an increased mutation load in the TME. We found that CYT-high HCCs were associated with significantly higher expressions of APOBEC3 family members (Figure 1a). The APOBEC3 score was generated by taking the log average expression in transcripts per million (TPM) of the following molecules: APOBEC3A, APOBEC3B, APOBEC3C, APOBEC3D, APOBEC3F, APOBEC3G, and APOBEC3H. This score was also elevated in CYT-high tumors (Figure 1b; *p* < 0.001). Furthermore, we investigated the mutation load in the TME. Interestingly, contrary to our hypothesis, the mutation load was not different between CYT-high HCCs and CYT-low HCCs (Figure S1d; *p* = 0.40). This similar mutation load between the groups can be due to a relatively low overall mutation load in HCC [10]. Similar findings were observed in pancreatic cancer as well [20]. CYT-high tumors had significantly less somatic copy number alterations (SCNAs) than the CYT-low group (Figure 1c; *p* = 0.003), mirroring the previous report. Davoli et al. reported that high levels of SCNAs, also known as aneuploidies, were found to correlate with aggressive tumor characteristics, such as enhanced cell cycle or cell proliferations, and reduce cytotoxic immune cell infiltrations [29].

### 2.3. CYT-High Tumors Have Significantly Higher Infiltration of Anticancer Immune Cells and Lower Infiltration of Procancer Immune Cells

**Figure 2:**
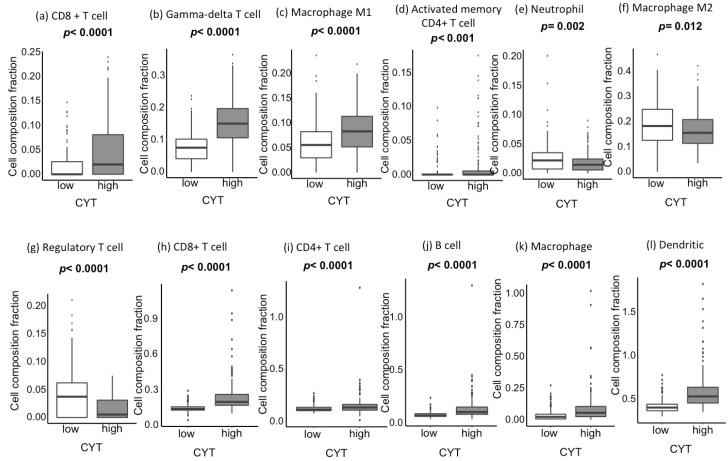
Association between the CYT and tumor-infiltrating immune cell composition in CIBERSORT and Tumor Immune Estimation Resource (TIMER). (**a**–**g**) CYT-high (185 patients) vs. CYT-low (186 patients) in CIBERSORT. (**a**) CD8+ T-cell (*p* < 0.0001), (**b**) gamma-delta T-cell (*p* < 0.0001), (**c**) macrophage M1 (*p* < 0.0001), (**d**) activated memory CD4+ T-cell (*p* < 0.001), (e) neutrophil (*p* = 0.002), (**f**) macrophage M2 (*p* = 0.012), and (**g**) regulatory T-cell (*p* < 0.0001). (h)-(l) CYT-high vs. CYT-low in TIMER. (**h**) CD8+ T-cell (*p* < 0.0001), (**i**) CD4+ T-cell (*p* < 0.0001), (**j**) B-cell (*p* < 0.0001), (**k**) macrophage (*p* < 0.0001), and (**l**) dendritic (*p* < 0.0001). All comparisons were performed using the Mann-Whitney test. CYT, cytolytic activity score.

CIBERSORT analyses revealed that CYT-high tumors have a significantly high infiltration of anticancer immune cells, such as cytotoxic T cells (Figure 2a), gamma-delta T cells (Figure 2b), M1 macrophages (Figure 2c), and activated memory CD4+ T cells (Figure 2d). On the contrary, CYT-high tumors have a significantly low infiltration of procancer immune cells, such as neutrophils (Figure 2e), M2 macrophage (Figure 2f), and regulatory T cells (Figure 2g). These results were mirrored by Tumor Immune Estimation Resource (TIMER) analyses, which estimates limited types of immune cells compared to CIBERSORT but with better resolution, using RNA sequencing (RNA-seq) data in the TME. TIMER revealed that CYT-high HCCs have a significantly higher infiltration of various immune cells, including CD8+ T cells, CD4+ T cells, B cells, macrophages, and dendritic cells (*p* < 0.0001, Figure 2h–l). However, TIMER does not have an ability differentiate anticancer and procancer immune cell types, except CD8+ T cells.

### 2.4. CYT was Associated with Tumor-Infiltrating Lymphocyte Receptor Genetic Diversity

**Figure 3:**
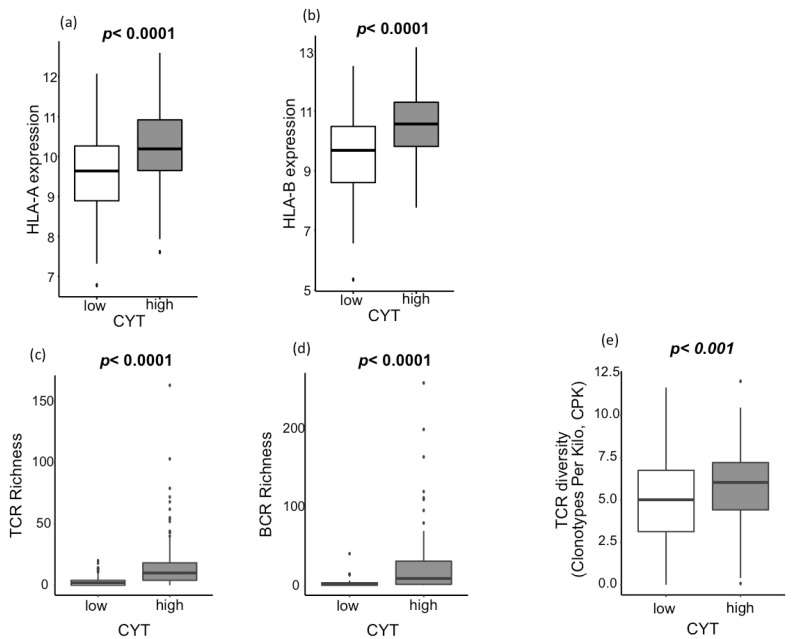
Association between the CYT and tumor-infiltrating lymphocyte receptor genetic diversity (**a**,**b**) Association between the CYT and human leucocyte antigen (HLA) expression. (**a**) Association between the CYT and HLA-A expression (*p* < 0.0001), and (**b**) association between the CYT and HLA-B expression (*p* < 0.0001). (**c**–**e**) Association between the CYT and lymphocyte receptor genetic diversity. (**c**) Association between the CYT and TCR richness (*p* < 0.0001), (**d**) association between the CYT and BCR richness (*p* < 0.0001), and (**e**) association between the CYT and TCR diversity (*p* < 0.001). CYT-high patients were 185 patients, and CYT-low were 186 patients. All comparisons were performed using the Mann-Whitney test. CYT, cytolytic activity score; TCR, T-cell receptor; BCR, B-cell receptor; and CPK, clonotypes per kilo.

The other components of cellular immunity other than infiltrating immune cells were analyzed. Expressions of human leucocyte antigen (HLA)-A and HLA-B, key molecules of major histocompatibility complex (MHC)-I, were significantly high in CYT-high HCCs (*p* < 0.0001, Figure 3a,b). Antigen-specific T cell receptor (TCR) and B cell receptor (BCR) repertoires are also important features of the immune system for the recognition of malignant cells and pathogens. We found that CYT-high HCCs associated significantly with the elevation of TCR richness, BCR richness, and TCR diversity (Figure 3c–e; *p* < 0.0001, *p* < 0.0001, and *p* = 0.0002, respectively). In order to further assess its clinical relevance, the impact of TCR richness on survival in HCC was further investigated (Figure S2a–d). TCR-high tumor has significantly improved the progression-free interval (PFI) (*p* = 0.009; Figure S2a), disease-free interval (DFI) (*p* = 0.002; Figure S2b), and disease-specific survival (DSS) (*p* = 0.02; Figure S2c) without significant difference in the overall survival (OS) (*p* = 0.14; Figure S2d). Our results reflect the notion that the increased TCR richness enhances anticancer immunity, which reflected on the clinical outcome [30,31].

### 2.5. CYT was Associated with the Expression of T Cell Exhaustion Markers

**Figure 4:**
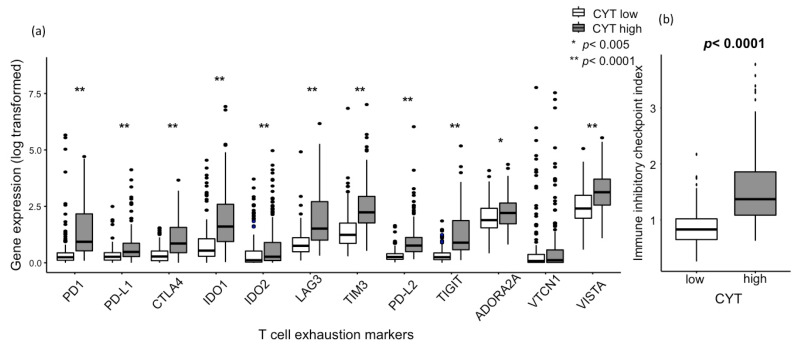
Association between the CYT and gene expression of regulatory T-cell markers. (**a**) Association between the CYT and major T cell exhaustion marker gene expressions, including PD-1 (*p* < 0.0001), PD-L1 (*p* < 0.0001), CTLA4 (*p* < 0.0001), IDO1 (*p* < 0.0001), IDO2 (*p* < 0.0001), LAG3 (*p* < 0.0001), TIM3 (*p* < 0.0001), PD-L2 (*p* < 0.0001), TIGIT (*p* < 0.0001), ADORA2A (*p* < 0.005), and VISTA (*p* < 0.0001). (**b**) Association between the CYT and immune inhibitory checkpoint index, generated from the key T cell exhaustion marker expression (*p* < 0.0001). CYT-high patients were 185 patients, and CYT-low were 186 patients. All comparisons were performed using the Mann-Whitney test. CYT, cytolytic activity score; PD-1, programmed death protein-1; PD-L1, programmed death-ligand 1; CTLA4, cytotoxic T-lymphocyte-associated protein 4; IDO1, indoleamine 2,3-dioxygenase 1; IDO2, indoleamine 2,3-dioxygenase2; LAG3, lymphocyte-activation gene3; TIM3, T cell immunoglobulin and mucin domain 3; PD-L2, programmed death-ligand 2; TIGIT, T cell immunoreceptor with Ig and ITIM domains; ADORA2A, adenosine A2a receptor; and VISTA, V-domain Ig suppressor of T cell activation.

T cells exhaustion markers are known as “brakes to the T cells”. We found that almost all the major T cell exhaustion markers were expressed significantly higher in CYT-high HCC, which include PD-1, PD-L1, CTLA4, indoleamine 2,3-dioxygenase 1 (IDO1), IDO2, lymphocyte-activation gene 3 (LAG3), T cell immunoglobulin and mucin domain 3 (TIM3), programmed death-ligand 2 (PD-L2), T cell immunoreceptor with Ig and ITIM domains (TIGIT), adenosine A2a receptor (ADORA2A), and V-domain Ig suppressor of T cell activation (VISTA) (Figure 4a). The immune inhibitory checkpoint index, generated from the key T cell exhaustion marker expression, was strongly elevated in CYT-high HCC (*p* < 0.0001, Figure 4b).

### 2.6. Gene Set Enrichment Analysis (GSEA) Demonstrated That Immune Response-Related Gene Sets were Significantly Enriched in CYT-High Hccs

As CYT-high HCCs have demonstrated stronger immunogenicity, we performed a GSEA to study the underlying mechanism. As demonstrated in Table 2, CYT-high HCCs were significantly associated with immune-response related gene sets, such as a defense response (normalized enrichment score, NES = 2.607; *p* < 0.0001), immune response (NES = 2.565; *p* < 0.0001), inflammatory response (NES = 2.562; *p* < 0.0001), immune system process (NES = 2.553; *p* < 0.0001), T-cell activation (NES = 2.139; *p* < 0.0001), and innate immune response (NES = 1.892; *p* = 0.004). These results suggest that an active crosstalk between innate and adaptive immunity is taking place in the CYT-high HCCs, and immunogenic HCCs can be appropriately selected by using the CYT. 

### 2.7. Patients with CYT-High HCCs Have Significantly Longer PFI and DFI and Better Prognosis than Patients with CYT-low HCCs

**Figure 5:**
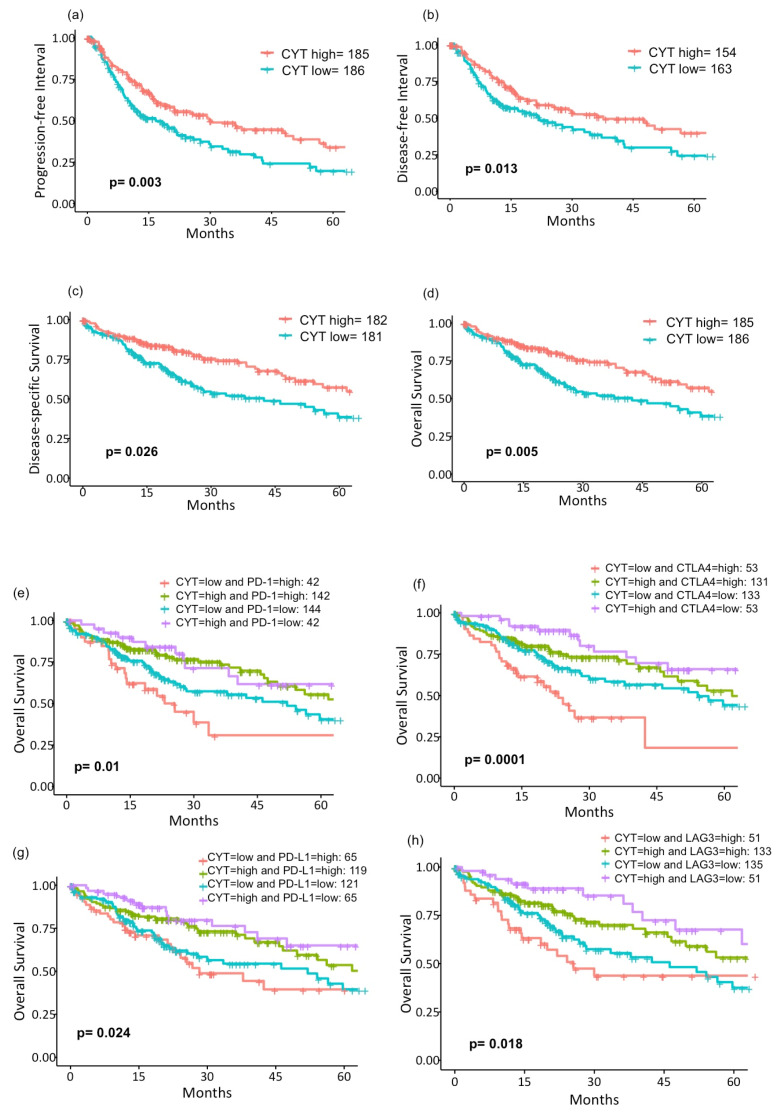
Survival analysis of the cancer genome atlas (TCGA) liver hepatocellular carcinoma (LIHC) patients. Kaplan-Meier curves of (**a**) PFI (*p* = 0.003), (**b**) DFI (*p* = 0.013), (**c**) DSS (*p* = 0.026), and (**d**) OS (*p* = 0.005) in CYT-high vs. -low patients. Survival curves of OS were further stratified by four major T cell exhaustion markers using the median value of each expression. (**e**) PD-1 (*p* = 0.01), (**f**) CTLA4 (*p* = 0.0001), (**g**) PD-L1 (*p* = 0.024), and (**h**) LAG3 (*p* = 0.018). Log-rank test was used to test group differences in survival analyses. CYT, cytolytic activity score; PFI, progression-free interval; DFI, disease-free interval; DSS, disease-specific survival; OS, overall survival; PD-1, program death-1; CTLA4, cytotoxic T-lymphocyte-associated protein 4; PD-L1, program death-ligand 1; and LAG3, lymphocyte-activation gene 3.

Due to the favorable tumor immune microenvironment of CYT-high tumors with the higher tumor killing activity, we hypothesized that CYT-high tumors would have better survival. We found that CYT-high tumors have significantly longer PFI (median interval: 30.4 months, 95% CI (21.2–56.7), *p* = 0.003; Figure 5a) and DFI (median interval: 37.7 months, 95% CI (25.1– not reached (NR)), *p* = 0.013; Figure 5b), as well as better DSS (median survival: 84.4 months, 95% CI (70.5–NR), *p* = 0.026; Figure 5c) and OS (median survival: 81.9 months, 95% CI (54.1–NR), *p* = 0.005; Figure 5d). On multivariate analysis, high CYT remained one of significant prognostic factors for the survival of patients with HCC (Table 3). Interestingly, with further stratification based on four major T cell exhaustion marker (PD-1, CTLA4, PD-L1, and LAG3) expressions dichotomized by the median value of each marker, CYT-low/exhaustion-high groups have the worst and CYT-high/exhaustion-low groups have the best overall survivals (Figure 5e–h). We further compared OS stratified by the CYT and TCR. Patients with higher CYT demonstrated significantly longer OS regardless of TCR, most likely due to a significant correlation between the CYT and TCR *(p* = 0.014; Figure S3).

## 3. Discussion

In the present study, we found that HCCs with high cytotoxic activity were significantly associated with high expressions of APOBEC3 genes with an increased infiltration of immune cells, such as CD8+ T cell, gamma-delta T cells, anticancer M1 macrophage, and dendritic cells, as well as with more diverse TCR and BCR repertoires. Due to strong immunogenicity and favorable immune microenvironments, CYT-high HCCs demonstrated significantly longer PFI, DFI, DSS, and OS compared to CYT-low HCCs. 

As shown by GSEA, this improved clinical outcome is speculated to be the result of a vigorous immune response generated in CYT-high tumors. This is in agreement with the high infiltration of anticancer immune cells, as well as significantly elevated expressions of T cell exhaustion markers in these tumors. With these results, we collectively speculated that an active crosstalk between innate and adaptive immune responses is taking place in the TME of CYT-high HCCs. In CYT-high HCCs, abundant macrophages and dendritic cells are present, creating “hot” TME, which attracts various lymphocytes. As demonstrated in other cancer types, such as melanoma or colorectal cancers, the higher infiltration of TILs often correlates with better clinical outcomes [30,32,33]; hence, CYT-high HCCs demonstrated better survival. Immunosuppressive mechanisms, driven by the immune system rather than cancer cells, were also observed, which are likely intrinsic as a negative feedback loop for CD8+ T cells, in line with other reports [20,34,35]. In order to further elucidate the immunosuppression effect, we further stratified the survival curve based on the CYT expression and four major T cell exhaustion markers (PD-1, PD-L1, CTLA4, and LAG3). Although major exhaustion marker expressions were found to be higher in CYT-high HCCs, a subset of patients with low CYT and high exhaustion marker HCCs demonstrated the worst prognosis. This finding likely resulted from its increased immunosuppressive mechanism combined with low cytolytic activity on this group. In contrast, patients with strong cytolytic activity and low exhaustion marker HCCs demonstrated the best clinical outcome. Similar to our findings, it has been reported that the ratio of regulatory T cells to cytotoxic T cells, and not simply the number of regulatory or cytotoxic T cells alone, resulted in poor prognosis in HCC [36,37].

In order to elucidate this finding, we further dissected the immune landscape of the TME, utilizing the CYT. The diversity of T cell and B cell repertoires are closely related to the capacity for lymphocytes to recognize antigens. This was reflected by our finding of an association between increased TCR repertoires with better clinical outcomes. Li et al. revealed that TCR diversity correlates with an enhanced immune response with a new mechanism to generate TCR diversity [30]. Their report supports our results that TCR and BCR diversity were significantly elevated in CYT-high HCCs, indicating that the cytotoxic activity is enhanced by more robust interaction between tumors and lymphocytes that take place in the TME. Furthermore, diversity in lymphocyte receptors is often positively correlated with increased neoantigen loads [30], part of which can be generated by the APOBEC3 family, major endogenous mutational sources in cancer 8. These previous reports advocate our result that there was a positive correlation between high CYT and the gene expression of each APOBEC3 member, as well as the APOBEC3 score [8,9,38]. Although the mutation load was not significantly different between the CYT-high and CYT-low HCCs, likely due to the overall low mutation burden in HCCs 10, anticancer immune cells were attracted to the TME of CYT-high HCCs. As the CYT is a mean expression value of GZMA and PRF1, it does not reflect the exact function of each immune cell. Rather, the CYT measures the overall immunogenicity of the TME. 

Since recent clinical trials with ICIs on HCC revealed less than a 20% objective response rate with a slight association with tumor cell PD-L1 expression [3,18,19], there is an imminent need for new biomarkers to identify respondents for immunotherapy. Recently, a quarter of the analyzed HCC cohort was found to have a significant enrichment of immune cell signatures and high PD-1/PD-L1 expressions [7]. Although this group of patients shared a strong immune cell infiltration in HCCs, two distinct groups were further identified with careful dissection of the gene expression profile in the TME: one characterized by the expression of adaptive immune response genes and another by the immunosuppressive signals [7]. Taken together with our results, subcategorizing tumors based on the immune landscape, such as the CYT and T cell exhaustion marker expressions beyond PD-L1, is expected to allow a precise patient selection for immunotherapy, including ICIs. Additionally, exploring the tumor immune microenvironment of HCC and deepening our understanding would further facilitate the development of new immunotherapy, as well as the establishment of a new treatment algorithm for HCC, beyond the current ones based upon tumor burden and underlying liver function [4,7,39,40]. We find our study novel, since the CYT represents tumor biology extremely well and would potentially serve as a possible biomarker for ICIs.

Despite these exciting results, the present study is not free from limitations. This study was conducted entirely using the single publicly available TCGA dataset without second validation cohorts due to their unavailability. Despite its significant benefits with clinicopathological data, along with gene expression information, TCGA has a few disadvantages as well. This study was based on the gene expression of the solely surgically resected primary tumor; thus, the role of the CYT is unclear in metastatic sites. Additionally, some clinical variables contained missing values in the cohort, including the AJCC T category, (*N* missing = 3), AJCC N category (*N* missing = 118), pathological stage (*N* missing = 24), adjacent hepatic tissue inflammation extent (*N* missing = 137), and underlying liver disease (*N* missing = 18). Additionally, the TCGA LIHC cohort did not include treatment information. Yet, our results suggested that the biology of CYT-high HCCs is significantly different from CYT-low HCCs, despite variable treatments. Lastly, this study does not include any in vitro or in vivo experiments; therefore, all our findings are based on exclusively association, although the concepts and mechanisms of the CYT have already been proved by the previous paper [23]. In order to prove the role of the CYT in HCCs, however, the experimental analyses, such as measurements of the CYT in the prospective cohort, will be required. On the other hand, while experiments are important, we also believe that it is as important to analyze the association in the large clinical cohort in order to prove the clinical relevance of the concept; hence, we conducted this study using the TCGA cohort.

## 4. Material and Methods

### 4.1. Patient Cohort and Genomic Data Processing

Both clinical and genomic data were obtained through the TCGA LIHC cohort. Clinical and copy number information were downloaded from cBioportal. Gene expression and mutation data were downloaded from the GDC legacy archive. In order to calculate the CYT, we used the transcripts per million (TPM) format of gene expression data, which is only available from the GDC portal. The RNA-seq data preprocessing and normalization method was performed as described in TCGA pipeline (https://docs.gdc.cancer.gov/Data/Bioinformatics_Pipelines/Expression_mRNA_Pipeline/). After excluding samples without gene expression data, 371 patients were utilized in the present study. There were missing clinical information in some of patients in the TCGA cohort, and we excluded them in the related analyses. As all patient information in the TCGA cohort were de-identified, the institutional review board (IRB) review was exempted. 

### 4.2. Cytolytic Activity Score (CYT)

CYT was defined as the geometric mean of GZMA and PRF1 expression values in TPM, as described previously [20,23,26,27] The threshold of dichotomization of the CYT-high and low groups was determined by the median of the CYT. There were 185 CYT-high patients and 186 CYT-low patients.

### 4.3. CIBERSORT and Tumor Immune Estimation Resource (TIMER)

CIBERSORT and Tumor Immune Estimation Resource (TIMER) were used to estimate infiltrating immune cell composition in the TME. CIBERSORT and TIMER calculate the fraction of 22 and 8 immune cell types, such as CD8+ T cells, CD4+ T cells, B cells, macrophage M1, M2, or regulatory T cells, using RNA-seq data in each tumor tissue via online calculators (https://cibersort.stanford.edu/ and https://cistrome.shinyapps.io/timer/) [41,42,43]. Given that TIMER only calculates 8 immune cell types compared to CIBERSORT, it does not have ability to differentiate the specific immune cell types. Each immune cell fraction was compared between CYT-high (185 patients) and low tumors (186 patients) in the TCGA LIHC cohorts with the Mann-Whitney test.

### 4.4. Gene Set Enrichment Analysis (GSEA)

Gene set enrichment analysis (GSEA) was performed between the CYT-high (185 patients) and CYT-low (186 patients) groups utilizing the annotated gene sets with the software (JAVA version 4.0) provided by the Broad Institute (https://software.broadinstitute.org/gsea/index.jsp), as described before [44,45,46,47,48]. Several collections of gene sets are available in the Molecular Signatures Database (MSigDB) (http://software.broadinstitute.org/gsea/msigdb) [49]. The primary result of the GSEA is the enrichment score (ES) and normalized enrichment scores (NES), which mirror that a certain gene set is enhanced at the top or bottom of the listed genes.

### 4.5. T cell receptor (TCR) Richness, B cell Receptor (BCR) Richness, and TCR Diversity

TCR richness and BCR richness values were collated from the Pan-Cancer Atlas study of Thorsson et al. [50]. TCR diversity was measured by using the number of unique complementarity determining region 3 (CDR3) cells in each sample divided by the total read count in the TCR region, which was represented as clonotypes per kilo reads (CPK) [30]. HLA-A and HLA-B are the expression levels of the HLA-A/B genes. The somatic copy number alteration (SCNA) event was defined by taking the sum of the segment mean changes ≥ 0.6 or ≤ -0.4 between the somatic and normal samples [20]. Each score was compared between CYT-high (185 patients) and low tumors (186 patients) with the Mann-Whitney test.

### 4.6. Statistical Analysis

Progression-free interval (PFI) was defined as the time between the date of diagnosis and the date of progression of HCC, disease-free interval (DFI) as the time between date of diagnosis and the date of diagnosis of a recurrent HCC, disease-specific survival (DSS) as the time from the date of diagnosis to the date of death by HCC, and overall survival (OS) as the time from the date of diagnosis to the date of death by any cause. PFI, DFI, DSS, and OS were compared between the groups with high CYT and low. Kaplan-Meier plots were created to depict the survival differences. Similarly, PFI, DFI, DSS, and OS were compared between the groups with high TCR and low, after being dichotomized by the median values of the TCR.

Cox progression hazards model was used for multivariate analysis to provide hazard ratios (HRs) and 95% confidence intervals (CIs). We utilized age, gender, stage, viral infection status, adjacent hepatic inflammation status, TIL, and CYT to perform a univariate analysis for the OS. Variables with significance were then selected as covariates for multivariate analysis. The differences between the two groups were assessed using the Mann-Whitney test for continuous variables, and chi-square tests were used for categorical variables. A two-sided *p*-value < 0.05 was considered statistically significant. Statistical analyses were performed using R software (http:///www.r-project.org/) and Bioconductor (http://bioconductor.org/).

## 5. Conclusions

In conclusion, CYT-high HCC is associated with significantly improved survival with enhanced immunity and increased cytolytic activity by the CD8+ T cell and M1 macrophage. Our findings suggest that examining and identifying tumor immune microenvironments in the HCCs could stratify the patients for appropriate treatments based on their biology. 

## Figures and Tables

**Table 1:** Clinical demographics of TCGA cohorts.

Variables	CYT Low (186)	CYT High (185)	*p*-Value
Age	59.5 ± 13.9	59.4 ± 13.1	0.68
Gender (Male/Female)	123/63	127/58	0.68
AJCC T category (T1/T2/T3/T4) *	83/54/44/5	98/40/36/8	0.19
AJCC N category (N0/N1) *	127/3	125/1	0.64
Pathological Stage (I/II/III/IV) *	77/48/45/3	94/38/40/2	0.34
Adjacent hepatic tissue inflammation extent (None/Mild/Severe) *	64/50/8	53/49/10	0.66
Underlying liver disease (No history/Alcohol/Hep B/Hep C/Others) *	56/52/36/16/16	35/65/44/18/14	0.12
TIL (Positive/Negative)	71/115	114/71	<0.001

TCGA, the cancer genome atlas; CYT, cytolytic activity; AJCC, American Joint Committee on Cancer; TIL, tumor infiltrating lymphocytes; Hep B, hepatitis B; and Hep C, hepatitis C. * Some of the clinical parameters were missing.

**Table 2:** Gene set enrichment analysis (GSEA) with immune response-related gene sets.

Gene set Name	ES	NES	*p*-Value
Defense Response	0.705	2.607	<0.001
Response To Wounding	0.636	2.565	<0.001
Immune Response	0.735	2.565	<0.001
Inflammatory Response	0.695	2.562	<0.001
Immune System Process	0.710	2.553	<0.001
Regulation Of Immune System Process	0.724	2.325	<0.001
Cellular Defense Response	0.789	2.302	<0.001
Leukocyte Activation	0.737	2.256	<0.001
Positive Regulation Of Immune System Process	0.724	2.229	<0.001
Humoral Immune Response	0.742	2.225	<0.001
Positive Regulation Of Cytokine Biosynthetic Process	0.739	2.139	0.001
T-Cell Activation	0.778	2.139	0.001
Cytokine Biosynthetic Process	0.631	2.100	0.001
Innate Immune Response	0.607	1.892	0.017

ES, enrichment score and NES, normalized enrichment score.

**Table 3:** Univariate and multivariate analysis for the OS.

Variables	Univariate Analysis			Multivariate Analysis		
	HR	95% CI	*p*-Value	HR	95% CI	*p*-Value
Age	1.03	1.005–1.049	0.016	1.03	1.006–1.051	0.013
Gender	0.81	0.483–1.364	0.432			
Stage (II vs. I)	1.83	0.948–3.538	0.072	2.03	1.046–3.948	0.036
Stage (III vs. I)	2.46	1.340–4.527	0.004	2.74	1.488–5.049	0.001
Stage (IV vs. I)	8.42	2.484–28.543	<0.001	12.58	3.531–44.853	<0.001
Adjacent hepatic tissue inflammation extent type (Mild vs. None)	1.24	0.717–2.129	0.446			
Adjacent hepatic tissue inflammation extent type (Severe vs. None)	0.98	0.343–2.778	0.964			
Virus status (Alcohol consumption vs. None)	0.58	0.291–1.175	0.132			
Virus status (Hepatitis B vs. None)	0.43	0.209–0.891	0.023			
Virus status (Hepatitis C vs. None)	1.05	0.424–2.602	0.916			
Virus status (Others vs. None)	0.94	0.400–2.210	0.887			
TIL (positive vs. negative)	1.11	0.662–1.864	0.692			
CYT (high vs. low)	0.55	0.326–0.921	0.023	0.47	0.275–0.819	0.007

OS, overall survival; HR, hazard ratio; CI, confidence interval; and CYT, cytolytic activity.

## Supplementary Materials

The following are available online at https://www.mdpi.com/2072-6694/12/5/1221/s1: Figure S1: (a) Expression of GZMA and PRF1 in HCC: Normal distribution of cytolytic genes within the TCGA LIHC cohort. (b) CYT expression in 50 matched samples of liver cancer and normal liver (*p* = 0.019, the Wilcoxon signed rank test). (c) The boxplot depicting the CYT among the groups of adjacent hepatic tissue inflammation extent (*p* = 0.352, Kruskal Wallis test). (d) Mutation load between the CYT-high (185 patients) and CYT-low (186 patients) groups (*p* = 0.40, Mann-Whitney test), Figure S2: Survival analysis stratified by TCR (cut-off at the median value) in the TCGA LIHC patients, Kaplan-Meier curves of (a) PFI (*p* = 0.009), (b) DFI (*p* = 0.002), (c) DSS (*p* = 0.02), and (d) OS (*p* = 0.14) in TCR-high vs. -low patients. Log-rank test was used to test group differences in the survival analysis, Figure S3: Survival analysis of the TCGA LIHC patients. Kaplan-Meier curve of OS stratified by the CYT and TCR (cut-off at the median value) (*p* = 0.014). Log-rank test was used to test group differences in the survival analysis.
