# Supplementary material for: Immune Cytolytic Activity for Comprehensive Understanding of Immune Landscape in Hepatocellular Carcinoma
Source: Cancers (Basel). 2020 May 13;12(5):1221. doi: 10.3390/cancers12051221 (PMC7281225; doi:10.3390/cancers12051221)
Supplement: Supplementary file 1 [file cancers-12-01221-s001.pdf]

cancers

MDPI

Supplementary Materials

Immune Cytolytic Activity for Comprehensive Understanding of Immune Landscape in Hepatocellular Carcinoma

Hideo Takahashi, Tsutomu Kawaguchi, Li Yan, Xuan Peng, Qianya Qi, Luc G.T. Morris, Timothy A. Chan, Allan Tsung, Eigo Otsuji and Kazuaki Takabe

Supplementary Materials:

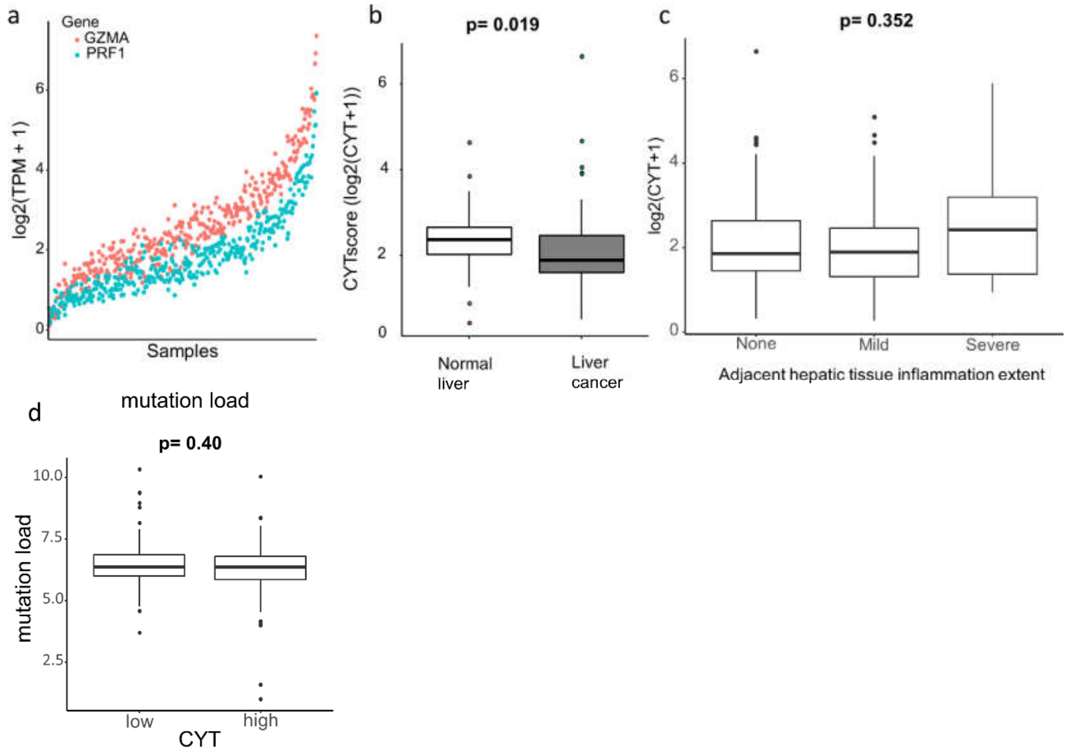

Figure S1. (a) Expression of GZMA and PRF1 in HCC: Normal distribution of cytolytic genes within the TCGA LIHC cohort. (b) CYT expression in 50 matched samples of liver cancer and normal liver (p = 0.019, the Wilcoxon signed rank test). (c) The boxplot depicting the CYT among the groups of adjacent hepatic tissue inflammation extent (p = 0.352, Kruskal Wallis test). (d) Mutation load between the CYT-high (185 patients) and CYT-low (186 patients) groups (p = 0.40, Mann-Whitney test).

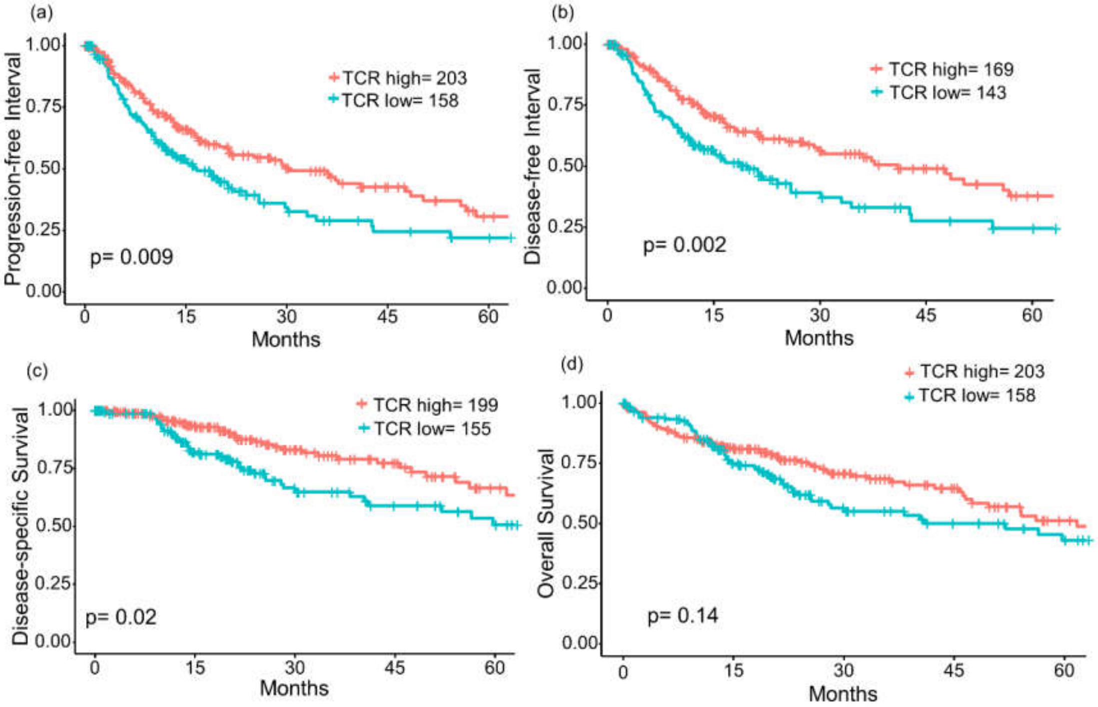

Figure S2. Survival analysis stratified by TCR (cut-off at the median value) in the TCGA LIHC patients, Kaplan-Meier curves of (a) PFI ($p = 0.009$), (b) DFI ($p = 0.002$), (c) DSS (p=0.02), and (d) OS ($p = 0.14$) in TCR-high vs. -low patients. Log-rank test was used to test group differences in the survival analysis.

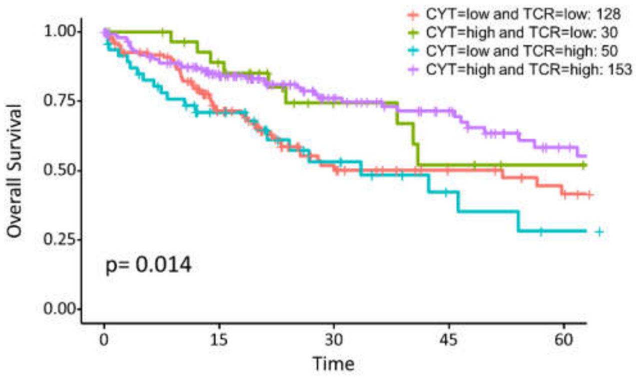

Figure S3. Survival analysis of the TCGA LIHC patients. Kaplan-Meier curve of OS stratified by the CYT and TCR (cut-off at the median value) ($p = 0.014$). Log-rank test was used to test group differences in the survival analysis.
